# *Streptococcus agalactiae* amylomaltase offers insight into the transglycosylation mechanism and the molecular basis of thermostability among amylomaltases

**DOI:** 10.1038/s41598-021-85769-3

**Published:** 2021-03-24

**Authors:** Suthipapun Tumhom, Pitchanan Nimpiboon, Kittikhun Wangkanont, Piamsook Pongsawasdi

**Affiliations:** 1 Starch and Cyclodextrin Research Unit, Department of Biochemistry, Faculty of Science, Chulalongkorn University, Bangkok, 10330 Thailand; 2 Center of Excellence for Molecular Biology and Genomics of Shrimp, Department of Biochemistry, Faculty of Science, Chulalongkorn University, Bangkok, 10330 Thailand; 3 Molecular Crop Research Unit, Department of Biochemistry, Faculty of Science, Chulalongkorn University, Bangkok, 10330 Thailand

**Keywords:** X-ray crystallography, Biocatalysis, Carbohydrates, Enzyme mechanisms, Enzymes, Proteins, Structural biology

## Abstract

Amylomaltase (AM) catalyzes transglycosylation of starch to form linear or cyclic oligosaccharides with potential applications in biotechnology and industry. In the present work, a novel AM from the mesophilic bacterium *Streptococcus agalactiae* (*Sa*AM), with 18–49% sequence identity to previously reported AMs, was characterized. Cyclization and disproportionation activities were observed with the optimum temperature of 30 °C and 40 °C, respectively. Structural determination of *Sa*AM, the first crystal structure of small AMs from the mesophiles, revealed a glycosyl-enzyme intermediate derived from acarbose and a second acarbose molecule attacking the intermediate. This pre-transglycosylation conformation has never been before observed in AMs. Structural analysis suggests that thermostability in AMs might be mainly caused by an increase in salt bridges since *Sa*AM has a lower number of salt bridges compared with AMs from the thermophiles. Increase in thermostability by mutation was performed. C446 was substituted with A/S/P. C446A showed higher activities and higher *k*_*cat*_*/K*_*m*_ values for starch in comparison to the WT enzyme. C446S exhibited a 5 °C increase in optimum temperature and the threefold increase in half-life time at 45 °C, most likely resulting from H-bonding interactions. For all enzymes, the main large-ring cyclodextrin (LR-CD) products were CD24-CD26 with CD22 as the smallest. C446S produced more CD35-CD42, especially at a longer incubation time.

## Introduction

Several different enzymes involved in the synthesis and degradation of starch have been studied^1^. Most of them, based on the amino acid sequence homology, belong to the α-amylase family^2^. One group of enzymes in the family is 4-α-glucanotransferase (4-αGTase), which cleaves an α-1,4-linkage of the glucan donor and transfers the glycosyl moiety to the acceptor^3^. Amylomaltase (AM, EC 2.4.1.25) is an intracellular 4-αGTase that catalyzes the intermolecular (disproportionation, coupling, hydrolysis) and intramolecular (cyclization) transglycosylation reactions to yield new linear oligosaccharides and LR-CD or cycloamylose (CA) products, respectively^3^. LR-CDs are used as an artificial chaperone for protein refolding^4,5^. In addition, AM has demonstrated promising applications in starch processing leading to several functional products^6^. It has been used in the production of thermoreversible starch gel, commercially available at present, to replace gelatin in food products^7^. Potential use of AM in the synthesis of functional oligosaccharides, such as isomaltooligosaccharides^8^ and anticariogenic maltooligosaccharides, has been recognized^9^. These beneficial aspects have created interests in developing novel enzymes and improving AM through various strategies for targeted properties.

AM was first identified in *Escherichia coli* as a maltose inducible enzyme that involved in maltose metabolism^10^. *AM* gene was reported in many bacterial strains, including the thermophilic^11,12^, hyperthermophilic^13^ and mesophilic bacteria^14^; and also in plants, which is known as disproportionating enzyme or D-enzyme^15,16^. Most of the reported AMs are thermostable enzyme from thermophilic bacteria. The three dimensional structures of about ten AMs have been published, most of which are from thermophiles, such as *Thermus aquaticus*^17^, *Thermus brockianus*^18^, *Thermus thermophilus*^19^, and *Aquifex aeolicus*^20^; while only two are from mesophilic bacteria *Corynebacterium glutamicum*^21^ and *E. coli*^22^. The first crystal structure of *T. aquaticus* AM (*Ta*AM) in complex with 34-meric CA, a polymeric substrate, has recently been successfully achieved^5^.

Thermostability is a desired property for industrial enzymes. However, the molecular basis for thermostability in AM remains elusive. Direct comparison between AM structures is difficult due to the differences in their sizes. *Cg*AM (AM from *C. glutamicum*) and *Ec*AM (AM from *E. coli*), which belong to the higher molecular weight group (around 84 kDa), are not thermostable^14,22^; but AM from *Thermus*, which has lower molecular weight (around 57 kDa), is thermostable^23,24^. To properly understand the thermostability in AMs, a search for a mesophilic bacterium, which gives AM of a similar molecular weight to the *Thermus*, is required. From the genome sequence of the mesophilic *Streptococcus agalactiae*, we found the putative AM sequence of the desired size. This present work has reported, for the first time ever, on the structural and biochemical characterization, and the catalytic mechanism of the AM from *S. agalactiae* (*Sa*AM). Site-directed mutagenesis on a selected target residue possibly involved in enzyme thermostability was performed in an attempt to understand the molecular basis of thermostability in AMs.

## Results and discussion

In this study, the *SaAM* gene, that encodes amylomaltase from mesophilic *S. agalactiae*, was cloned, expressed, and characterized biochemically and structurally. Cysteine 446 was selected for mutagenesis experiment in an attempt to enhance enzyme thermostability. Amino acid compositions and bonding interactions of *Sa*AM in comparison to thermostable AMs were analyzed to better understand the rationale behind thermostability in AMs.

### Gene cloning and molecular characterization of *Sa*AM

**Figure 1:**
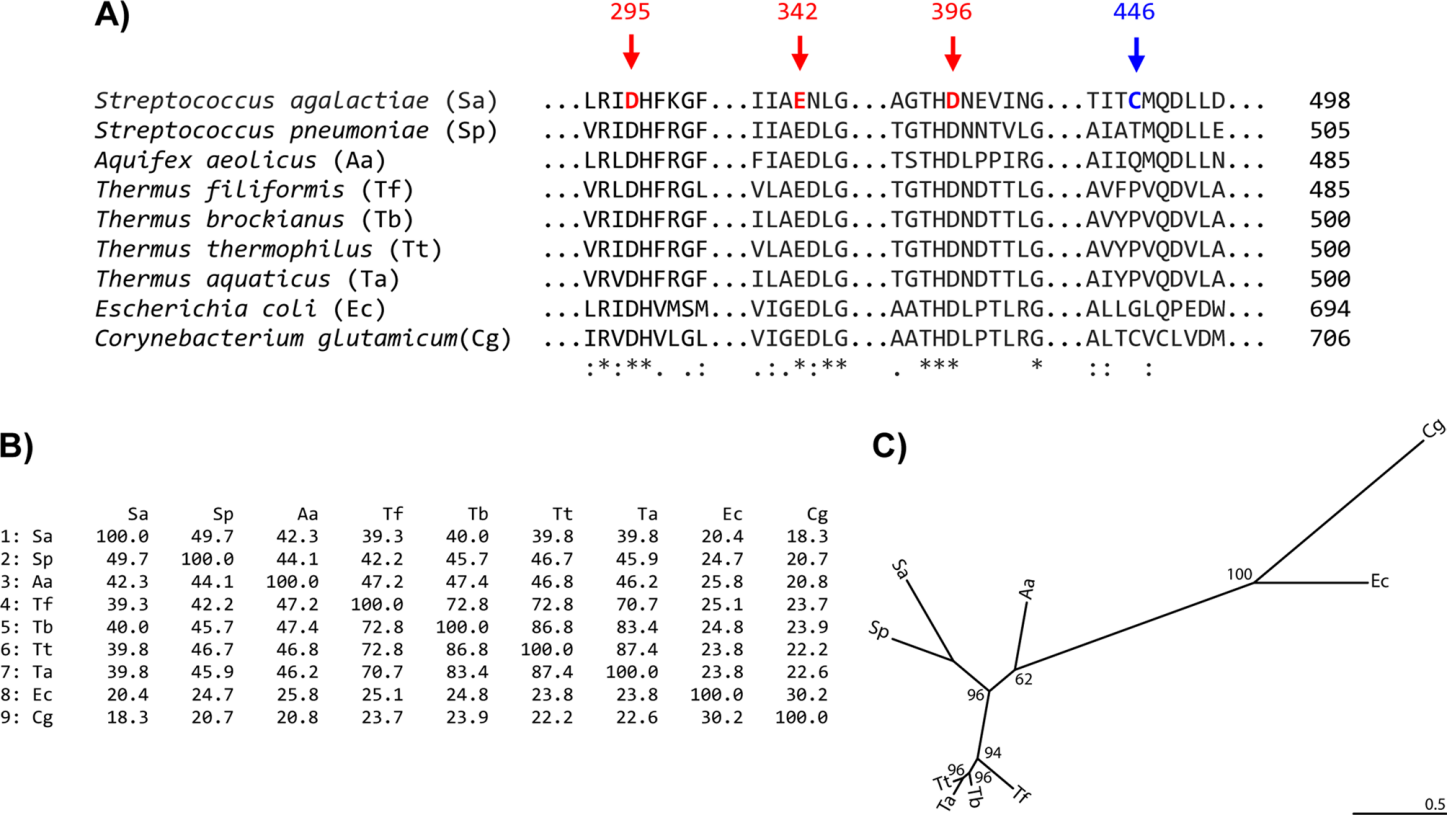
(**A**) Alignment of amino acid sequence of amylomaltases from various bacteria around the regions of interest. Sa; *Streptococcus agalactiae* (this study), Sp; *Streptococcus pneumoniae* (WP_00749291), Aa; *Aquifex aeolicus* (AAC06897), Tf; *Thermus filiformis* (AKR04336), Tb; *Thermus brockianus* (2X1I_A), Tt; *Thermus thermophilus* (YP_144527), Ta; *Thermus aquaticus* (EED09753), Ec; *Escherichia coli* (P15977), Cg; *Corynebacterium glutamicum* (NP_601497). The three catalytic residues are shown in red. C446 of Sa, the residue that was mutated in this study is shown in blue. Amino acid conservation across the aligned sequences is shown as: identical (*asterisk)*, conserved substitutions (*colon*) and semi-conserved substitutions (*dot*). The number at the end of each sequence denotes the total amino acid sequence length of each enzyme. (**B**) Amino acid identity matrix. (**C**) Unrooted phylogenetic created by IQ-TREE with bootstrap values at the nodes. Length scale is shown at the bottom-right corner.

When the putative 4-α-glucanotransferase gene of *S. agalactiae* (*SaAM*) was cloned in *E. coli*; the ORF of 1,494 bps encoding for 498 amino acid residues was obtained. The protein sequence was subjected to a BLAST search at the National Center of Biotechnology^25^ and the multiple sequence alignment was as shown (Fig. 1A). Our cloned sequence of *Sa*AM is identical to the sequence entry WP_000745455 deposited in GenBank; and the entry was derived by the automated computational analysis from the genome sequence of *S. agalactiae*^26^*. Sa*AM is a novel enzyme because it showed a low amino acid sequence identity with previously reported AMs, even with an AM of the same genus *S. pneumoniae*^27^, which showed only 49.7% amino acid sequence identity (Fig. 1B). Compared with thermostable AMs from *Thermus*, about 40.0% sequence identity was observed. Meanwhile very low homology with AMs from *E. coli* and *C. glutamicum* mesophilic bacteria with long N-terminus, was found^21,22^ with the identities of only 20.4% and 18.3%, respectively. However, when analyzed from the phylogenetic tree, *Sa*AM is grouped with AMs from other mesophilic bacteria, away from those AMs of the thermophiles (Fig. 1C). *Sa*AM has the conserved catalytic and substrate recognition residues (D295, E342 and D396, *Sa*AM numbering), which are found in all AMs. In addition, *Sa*AM contains the characteristic sequence corresponding to the (α/β)_8_ domain, with the three inserted subdomains, four conserved regions and two unique loops of GH77 family, which is similar to all other AMs^17,28^.

### Expression and purification of recombinant *Sa*AM

In the production of *Sa*AM, the recombinant cells were able to express high amount of crude enzyme in soluble fraction, when cultured at 37 °C under IPTG, with a specific activity of 5.8 U/mg protein (Table 1). The high intensity protein band was observed in SDS gel (Fig. S1). The crude *Sa*AM was purified by HisTrap FF column (Table 1). The apparent molecular mass of *Sa*AM, as determined by SDS-PAGE analysis, was 57 kDa (Fig. S1) with the calculated pI value of 4.80. When compared with other AMs, the size of *Sa*AM was close to AMs from thermophilic bacteria (52–55 kDa), such as *T. brockianus, T. thermophilus, T. aquaticus* (all consisting of 500 amino acid residues), and *T. filiformis* (485 residues)^18,24,29,30^. Besides, the size of *Sa*AM was close to plant D-enzymes (59 kDa), such as cassava *Manihot esculenta* Crantz (585 residues) and *Arabidopsis sp*. (576 residues)^16^. Interestingly, *Sa*AM showed significant difference in molecular mass when compared with AMs from the two mesophilic bacteria, *Ec*AM (694 residues, 78 kDa)^22^ and *C*gAM (706 residues, 84 kDa)^14^. However, a similar size enzyme containing 505 amino acid residues was found in the AM of *S. pneumoniae*, a mesophilic bacterium of the same genus^27^.

**Table 1:** Purification table of WT and C446 mutated *Sa*AMs.

	Purification step	Total protein	Total activity	Specific activity	Yield	Purification fold
		(mg)	(U)	(U/mg protein)	%	
WT	Crude extract	329	1915	5.8	100	1.0
	HisTrap FF™	21	1147	54.3	60	9.3
C446A	Crude extract	401	2109	5.3	100	1.0
	HisTrap FF™	23	1584	69.6	75	13
C446P	Crude extract	249	1537	6.2	100	1.0
	HisTrap FF™	17	846	51.1	55	8.3
C446S	Crude extract	386	1941	5.0	100	1.0
	HisTrap FF™	22	1313	59.7	68	12

Crude extract was prepared from 1.2 L of cell cultures (6.98 g cell wet weight).

### Structural characterization of WT *Sa*AM

**Figure 2:**
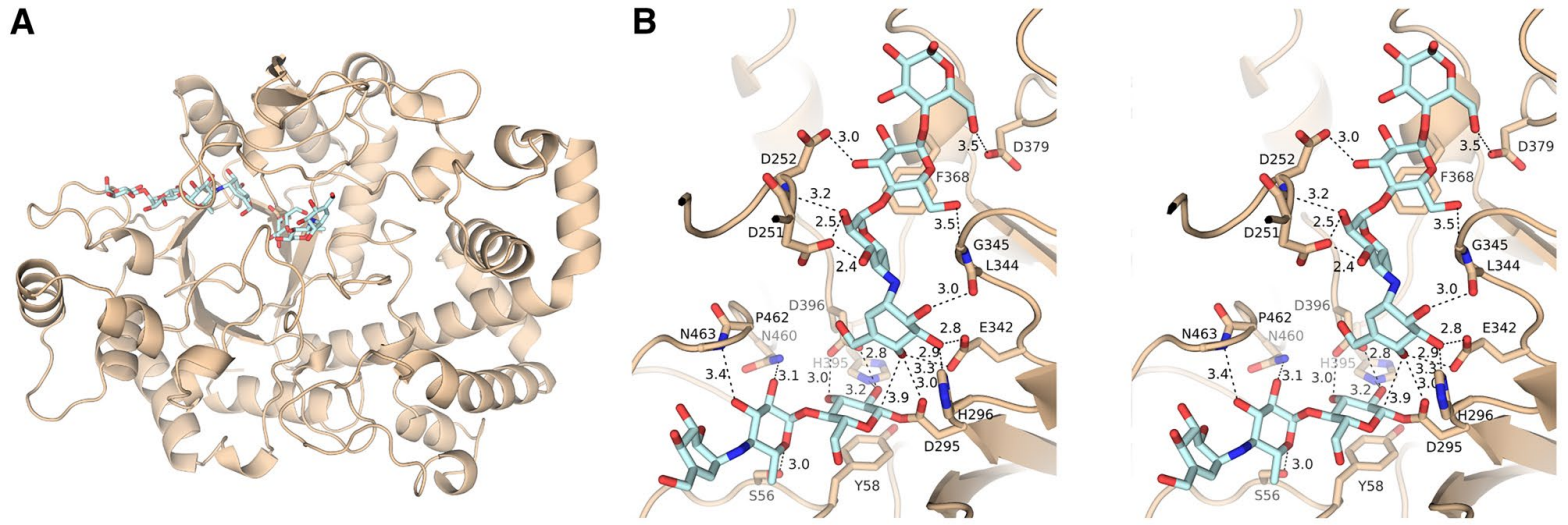
(**A**) Crystal structure of *Sa*AM (wheat) with acarbose and the acarbose-derived glycosyl-enzyme intermediate (cyan). (**B**) Stereo images of the *Sa*AM active site. Interactions are shown in dash lines with distances in angstrom (Å).

To date, three-dimensional structure of small AM from mesophilic bacteria is not yet available. Therefore, we determined the X-ray crystal structure of *Sa*AM in complex with acarbose to gain an insight into its thermostability and catalytic mechanism. The data reduction and refinement statistics are shown in Table 2. Eight chains of *Sa*AM were identified in the asymmetric unit, and the results showed that all chains are similar. Chain C, which has the best real-space correlation coefficient, was used for further analysis of *Sa*AM in complex with acarbose (Fig. 2A). The RMSD values of *Sa*AM is comparable to *T. brockianus* (PDB2X1I)^18^, *T. thermophilus* (PDB 2OWC)^19^, *T aquaticus* (PDB 1ESW)^29^, and *A. aeolicus* (PDB 1TZ7)^20^ AMs which are 0.788, 0.793, 0.770, and 0.748, respectively. Thus, *Sa*AM has similar overall three-dimensional structure compared with thermophilic AMs. We found two ligands present in the active site (Figs. 2B and S2). The first ligand is an acarbose molecule that had reacted with the catalytic nucleophile D295, cleaving off a glucose residue at the reducing end, resulting in an acarbose-derived glycosyl-enzyme intermediate at D295. The second ligand is an intact acarbose molecule close to the glycosyl-enzyme intermediate. The 4-OH group of the non-reducing end of the second acarbose molecule is around 3.9 Å away from the anomeric carbon atom of the glycosyl-enzyme intermediate. Therefore, we believe that we have captured the pre-transglycosylation step of the catalytic mechanism with the acarbose molecule resembling a non-reducing end attacking the glycosyl-enzyme intermediate. This catalytic conformation has not been reported before among AMs. The alkene moiety in the non-reducing end may have caused the 4-OH to be in a sub-optimal position for nucleophilic attack, thus allowing isolation of the catalytic intermediate. *Sa*AM also makes extensive contacts with both the glycosyl-enzyme intermediate and the attacking acarbose molecule. For the glycosyl-enzyme intermediate, the glucose residue covalently linked to D295 (D293, *Ta*AM numbering^29^) also interacts with H395 and the transition- state stabilizer D396 at 2-OH and 3-OH. H395 and D396 are conserved in all reported AMs; their numberings in *Ta*AM and *Cg*AM are H384 and H560, respectively^14,29^. The 4-amino-4,6-dideoxy-glucose residue interacts with *Sa*AM at the side chain of S56 and N460, as well as the NH group of the backbone peptide bond between P462 and N463. For the acarbose molecule, the 3-OH group of the non-reducing end is recognized by H296 and E342, the general acid–base catalysts. Both H296 and E342 are conserved residues in AMs with H296 corresponding to H294 of *Ta*AM and H461 of *Cg*AM^23,31^. The 2-OH forms a H-bond with the carbonyl group of the backbone peptide bond between L344 and G345. The interactions of the enzyme with the 2-OH and 3-OH groups of the non-reducing end help position the 4-OH for nucleophilic attack and also block the 2-OH and 3-OH groups from reacting with the glycosyl-enzyme intermediate. Thus, our novel structure also explains the regioselectivity of the transglycosylation reaction. Other interactions of the acarbose molecule with the enzyme include the side chain of D251, D252, and the peptide bond between these two residues. The NH group of the backbone peptide bond between L344 and G345 forms a bonding with the 6-OH of the second glucose residue of acarbose, the 6-OH of the first glucose residue of acarbose interacts with the side chain of D379.

**Table 2:** Data collection and refinement statistics for WT *Sa*AM.

Data collection statistics	PDB ID 6M6T
Wavelength (Å)	0.999999
Resolution range (Å)	29.82–2.75 (2.80–2.75)
Space group	P 2_1_ 2_1_ 2_1_
Unit cell dimensions	103.7 216.1 224.5
Total number of reflections	921,329 (37,365)
Number of unique reflections	129,800 (6,317)
Multiplicity	7.1 (5.9)
Completeness (%)	98.9 (98.1)
Mean I/σ(I)	10.5 (2.1)
Wilson B factor (Å^2^)	42.78
R_merge_	0.136 (0.840)
R_meas_	0.147 (0.924)
R_pim_	0.055 (0.377)
CC_1/2_	0.996 (0.745)

Refinement statistics	
Resolution range (Å)	29.82–2.75 (2.85–2.75)
R factor	0.2054 (0.2865)
R_free_ (5%)	0.2869 (0.3852)
**Number of atoms**	
Protein	32,590
Acarbose and derivatives	554
Water	252
**Number of protein residues**	
Chain A	496
Chain B	497
Chain C	499
Chain D	496
Chain E	497
Chain F	496
Chain G	498
Chain H	495
RMSD for bonds (Å)	0.010
RMSD for angles (deg)	1.128
Estimated coordinate error (ML, Å)	0.41
Ramachandran favored (%)	94.24
Ramachandran outliers (%)	0.00
Average isotropic B factor (Å^**2**^**)**	50.59
**Chain A**	
Protein	54.75
Glycosyl-enzyme intermediate	62.23
Acarbose in the transglycosylation site	76.82
Water	46.72
**Chain B**	
Protein	53.64
Glycosyl-enzyme intermediate	53.34
Acarbose in the transglycosylation site	63.26
Water	46.74
**Chain C**	
Protein	44.06
Glycosyl-enzyme intermediate	42.72
Acarbose in the transglycosylation site	55.07
Water	39.06
**Chain D**	
Protein	48.31
Glycosyl-enzyme intermediate	55.84
Acarbose in the transglycosylation site	73.72
Water	41.95
**Chain E**	
Protein	43.58
Glycosyl-enzyme intermediate	41.77
Acarbose in the transglycosylation site	57.46
Water	41.99
**Chain F**	
Protein	48.23
Glycosyl-enzyme intermediate	52.59
Acarbose in the transglycosylation site	73.45
Water	44.17
**Chain G**	
Protein	50.20
Acarbose by the catalytic residue	66.39
Acarbose in the transglycosylation site	74.94
Water	43.41
**Chain H**	
Protein	61.05
Glycosyl-enzyme intermediate	63.28
Acarbose in the transglycosylation site	68.97
Water	45.96
**Real-space correlation coefficient of ligands**	
**Chain A**	
Glycosyl-enzyme intermediate	0.88
Acarbose in the transglycosylation site	0.79
**Chain B**	
Glycosyl-enzyme intermediate	0.91
Acarbose in the transglycosylation site	0.87
**Chain C**	
Glycosyl-enzyme intermediate	0.95
Acarbose in the transglycosylation site	0.92
**Chain D**	
Glycosyl-enzyme intermediate	0.86
Acarbose in the transglycosylation site	0.88
**Chain E**	
Glycosyl-enzyme intermediate	0.91
Acarbose in the transglycosylation site	0.86
**Chain F**	
Glycosyl-enzyme intermediate	0.89
Acarbose in the transglycosylation site	0.82
**Chain G**	
Acarbose by the catalytic residue	0.89
Acarbose in the transglycosylation site	0.81
**Chain H**	
Glycosyl-enzyme intermediate	0.86
Acarbose in the transglycosylation site	0.86

Statistics for the highest-resolution shell are given in parentheses.

As mentioned above, the pre-transglycosylation intermediate was first seen in our crystal structure of *Sa*AM in complex with acarbose. In *Ta*AM, the first elucidated crystal structure of GH77, two acarbose molecules bound to the enzyme, one in the active site at substrate- binding subsites − 3 to + 1 and another at 14 Å away from the non-reducing end of the first acarbose^17,29^. Ligand interactions were identified, and the catalytic mechanism through the glycosyl-enzyme intermediate was supported. In the more recent study, the first AM structure from a mesophilic bacterium *E. coli*, *Ec*AM, with the N-terminal extension of about 140 residues in comparison with *Ta*AM, has been determined through the three crystal structures in the apo form and in complex with maltose substrate and acarbose inhibitor. The structure of acarviosine-glucose-acarbose, consisting of two acarbose molecules joined to obtain a heptasaccharide analog corresponding to the transglycosylation product, was captured^22^. The ability to detect the pre-transglycosylation intermediate in the present study gave a direct clue to the transglycosylation mechanism for AMs.

### Site-directed mutagenesis of *Sa*AM

From the observed 3D-structure of *Sa*AM, a single cysteine (C446) corresponds to the conserved proline (P450, *Ta*AM numbering) in all reported AMs from *Thermus*. In the *Cg*AM and *Ec*AM from mesophilic bacteria, the residues are C647 and G634, respectively (Fig. 1A). Since proline, a nonpolar aliphatic amino acid, is known to contribute to protein stability by decreasing the conformational freedom of the protein backbone^32,33^, and high amount of proline was observed in *Thermus* AM; thus, we hypothesize that changing C to P would enable *Sa*AM to work better at high temperature. In addition, mutations to serine (S, polar uncharged) and alanine (A, nonpolar aliphatic) were performed. Serine mutants have been found to increase thermostability in many enzymes due to its polar nature and ability to form H-bonding resulting in protein stabilization^34^; while alanine is nonpolar aliphatic, similar to proline but smaller. Hence, C446 of *Sa*AM was substituted by A/P/S. These mutations may shed lights to the understanding of control of thermostability in the AMs of thermophilic bacteria. The recombinant plasmid pET-28a containing *SaAM* gene was used as a template for site-directed mutagenesis. The result of mutation on *SaAM* gene was confirmed by nucleotide sequencing. The mutated *Sa*AMs were expressed and purified using the same protocol as for the WT. Then, they were biochemically characterized in comparison to the WT.

### Biochemical characterization of WT and C446 mutated *Sa*AMs

#### Amylomaltase activities

The intermolecular transglycosylation (starch transglycosylation, disproportionation, coupling, and hydrolysis) and intramolecular transglycosylation (cyclization) activities of WT and mutated *Sa*AMs were measured (Table 3). The results showed that WT enzyme had high starch transglycosylation, disproportionation and cyclization activities but low hydrolysis and coupling activities similar to all previously reported AMs^11,14,18^. Mutation at C446 caused an obvious change in enzyme activities, especially of the A mutant which showed a significant increase in three main activities of AM: starch transglycosylation, disproportionation and cyclization. However, C446P showed a slight reduction in those activities while relatively similar activity was observed for the S mutant.

**Table 3:** Activities of WT and C446 mutated *Sa*AMs.

Enzyme	Specific activities (U/mg protein)				
	Starch tranglycosylation	Disproportionation	Cyclization	Coupling	Hydrolysis
WT	57 ± 2	54 ± 1	0.9 ± 0.2	0.19 ± 0.01	0.05 ± 0.01
C446A	73 ± 1	70 ± 1	1.3 ± 0.2	0.16 ± 0.02	0.03 ± 0.01
C446P	46 ± 1	51 ± 1	0.7 ± 0.1	0.12 ± 0.01	0.02 ± 0.01
C446S	56 ± 1	60 ± 1	0.8 ± 0.1	0.15 ± 0.01	0.04 0.01

Data are shown as the mean ± standard deviation and are derived from three independent experiments.

#### Optimum conditions and thermostability

**Figure 3:**
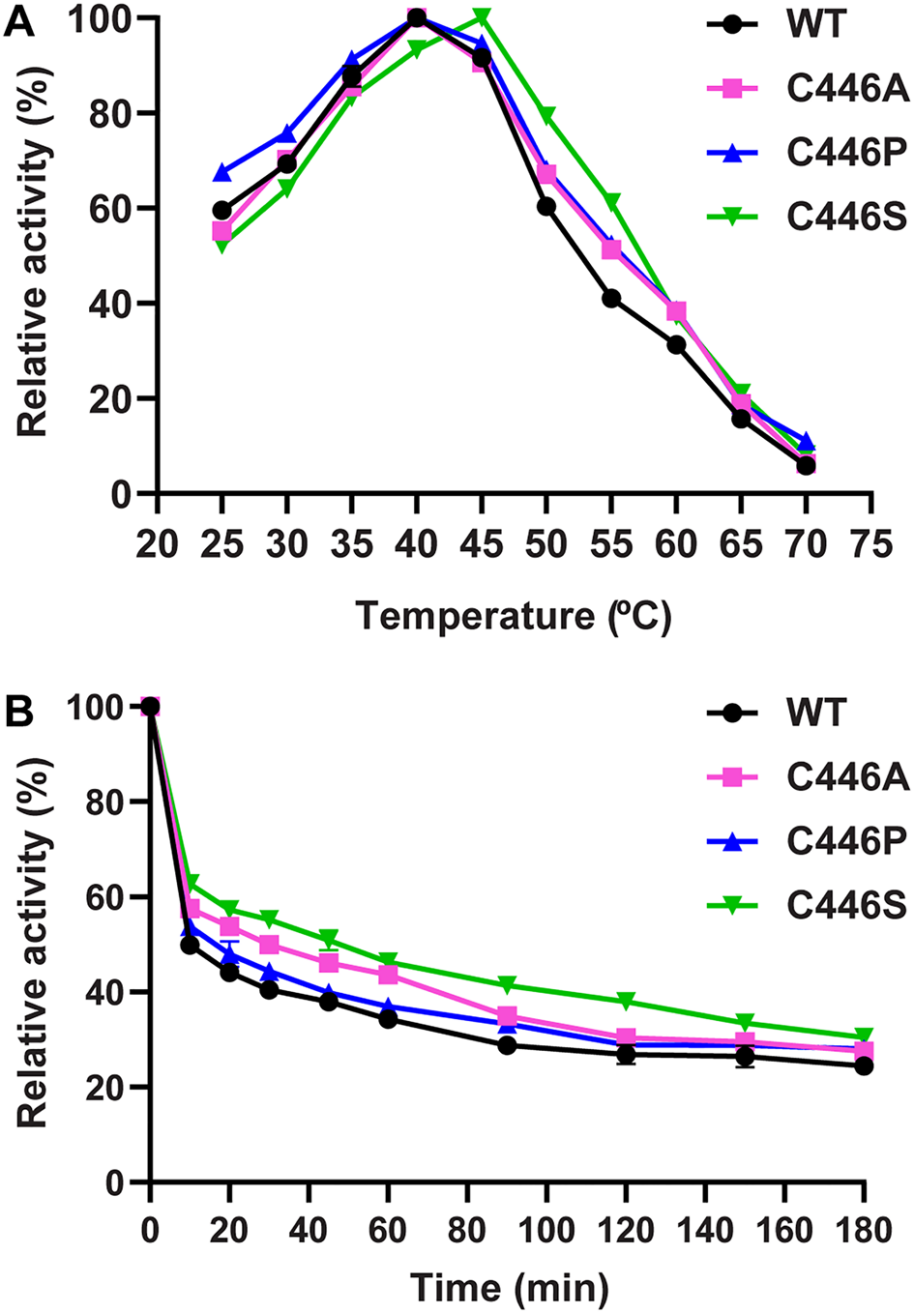
(**A**) Optimum temperature of WT and C446 mutated *Sa*AM. (**B**) Effect of temperature on *Sa*AM stability. WT and C446 mutated *Sa*AMs were incubated in phosphate buffer pH 6.0 at 45 °C from 0 to 180 min.

The optimum temperature for disproportionation reaction of WT *Sa*AM was at 40 °C (Fig. 3A), the same as *Cg*AM^14^ but different from *Ec*AM (35 °C)^22^. Meanwhile the AMs from the three thermophiles (*T. filiformis*, *Thermotoga maritima, T. aquaticus*) and the hyperthermophile *A. aeolicus* displayed the optimum temperatures of 60 °C, 70 °C, 75 °C, and 90 °C, respectively^11,12,24,35^. Interestingly, C446S-*Sa*AM had an optimum temperature of 45 °C, a 5 °C increase over that of the WT, while the optimum temperature of A and P mutants did not change (Fig. 3A).

The optimum pH of WT *Sa*AM was at pH 6.0, the values of *Cg*AM and those of *Thermus* are similar or more or less the same^14,36^. Mutation at C446 did not result in any change in optimum pH, suggesting that C446 was not involved in catalysis affected by functional group ionization.

For enzyme stability, the effect of temperature was determined by following the disproportionation activity of *Sa*AM as a function of time. The enzyme was pre-incubated at different temperatures ranging from 30 to 50 °C for 0–180 min as previously described^36^. Figure 3B displays enzyme stability at 45 °C. C446S showed a threefold improvement of half-life time compared with the WT. At all temperatures studied, we surprisingly found that stability of the P mutant was not much higher than the WT, and the S mutated enzyme was the most stable.

**Figure 4:**
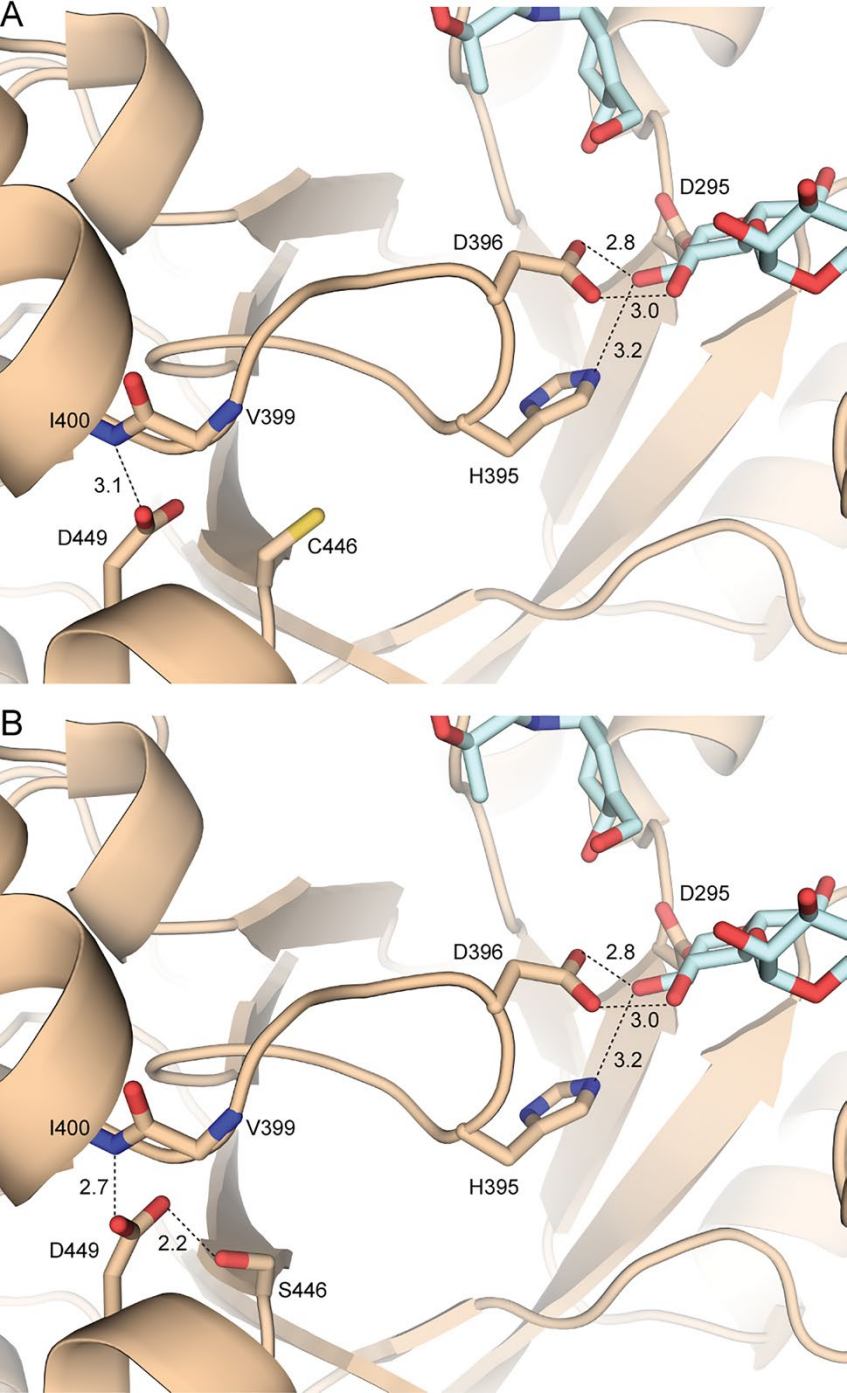
(**A**) Crystal structure of *Sa*AM (wheat) and carbohydrate residues (cyan) near C446. (**B**) Model of possible interactions when C446 is mutated to serine.

Because *Sa*AM contains a single cysteine residue (C446), we proposed that mutation of C446 to serine (C446S) will not only render reduction of oxidative damages to the protein, which is a favorable characteristic for further enzyme applications, but also increase possibility of forming H-bonds with nearby residues resulting in a favorable conformation for thermostability. The increase in thermostability is a major cause for an upward shift of the optimum temperature. Possible interactions through H-bonding are evidenced as shown in the crystal structures of the WT and the S mutant through structural modeling (Fig. 4). In the WT enzyme, C446 does not form any H-bond. However, the S mutant could potentially form a H-bond with the side chain of D449. The side chain of D449 can also make a H-bond with the main chain NH of I400 in the WT enzyme. Thus, S446 should hold the D449 side chain in place and help stabilize the interaction between D449 and I400. I400 is a part of the loop containing H395 and D396 that interact with the glycosyl-enzyme intermediate by H-bonds. Thus, we propose that C446S may stabilize the interactions between H395 and D396 and the substrate, and improve both the thermostability and the catalytic activity. H395 of *Sa*AM is corresponded to H394 of *Tt*AM, which was previously reported as a substrate-binding residue near the active site^23^.

Studies in different enzymes/proteins have shown similar results of serine replacement causing an increase in enzyme/protein stability. Substitution with polar amino acids in lysozyme led to an increase in protein stability through the creation of H-bonds^37^. In 2004, Kwa et al*.* reported that H-bond formed between serine and a keto group of bacteriochlorophyll protein increased thermal stability of the model protein complex in the native membrane^38^. The number of H-bonds between the side chains of amino acid residues was found to correlate with thermostability of α-amylase^39^. In AMs, a single substitution forming the A406V and N287Y mutated *Cg*AMs (the residues located around the second glucan binding site, 14 Å from the active site) and the mutant E27R at the surface of *Tf*AM were reported to increase enzyme thermostability and also cause an upward shift in optimum temperature^24,40^.

#### Substrate specificity and kinetic parameters

**Figure 5:**
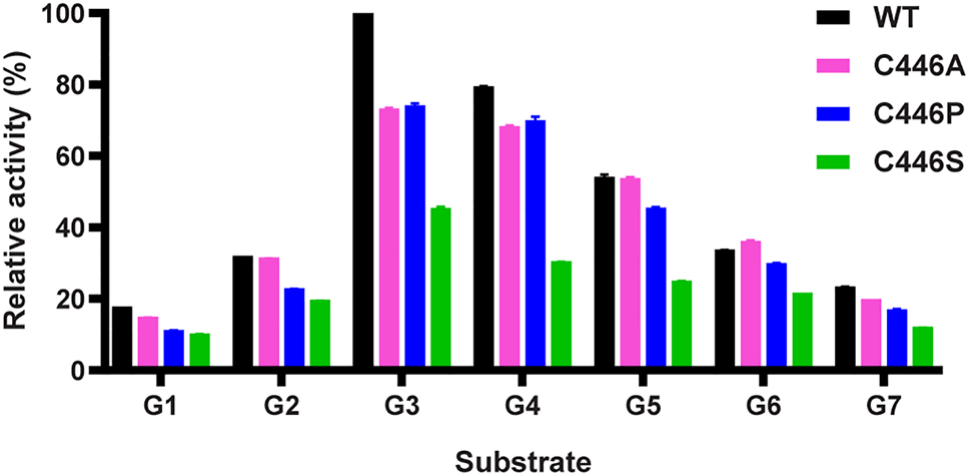
Substrate specificity of WT and C446 mutated *Sa*AMs in disproportionation reaction using malto-oligosaccharides (maltose, G2 to maltoheptaose, G7) as substrate. The activity of WT-*Sa*AM on G3 substrate was set as 100%. *P < 0.05 (Student’s t-Test with respect to the disproportionation of WT). Data are presented as the mean ± SD and are derived from three independent experiments.

The ability of *Sa*AM to utilize different substrates (G2-G7) in disproportionation reaction was determined (Fig. 5). The results showed that while maltotriose (G3) was the most efficient substrate, maltoheptaose (G7) was the poorest for both WT and C446 mutated *Sa*AMs. The order of preferred substrates of WT *Sa*AM was G3 > G4 > G5 > G6 > G2 > G7, which is in accordance with several WT AMs from thermophilic and mesophilic bacteria^11,14,22^. The highest specificity for G3 was reported in almost all AMs, including those from *Thermus *sp., *A. aeolicus*, *E. coli*, and *C. glutamicum*^11,12,14,22^. Moreover, G3 was also the best substrate for DPE1 from plants, such as potato and cassava^16^. The C446 mutants showed the same order of preferred substrates as the WT. On the other hand, the A and P mutants showed preferences for larger G4 to G6 and their specificities for G3 and G4 were almost the same. From these overall results, mutation at C446 residue of *Sa*AM did not cause any major change in substrate specificity.

To follow whether interactions between *Sa*AM and its substrates change upon mutation, kinetic analysis was performed on both the disproportionation and cyclization reactions. For disproportionation, the most favorable G3 substrate was used. The results indicated that C446A and C446S had *k*_*cat*_/*K*_*m*_ values close to that of the WT, while C446P gave somewhat lower value (Table 4A). However, all *Sa*AMs had similar *K*_*m*_ values in the range of 21–24 mM. This suggests that the mutation did not cause any obvious change in substrate (G3) binding and catalytic rate, which implies that not much of a change in enzyme conformation occurred.

**Table 4:** Kinetic parameters of WT and C446 mutated *Sa*AMs from disproportionation reaction (A) and cyclization reaction (B).

A				
*Sa*AM	*K*_*m*_ (mM)	*V*_*max*_ (µmole glucose min^−1^ µg protein^−1^)	*k*_*cat*_ (min^-1^) [10^3^]	*k*_*cat*_/*K*_*m*_ (mM^−1^ min^−1^) [10^3^]
WT	21 ± 3	2 ± 0.1	180 ± 2	9 ± 2
C446A	22 ± 2	3 ± 0.1	220 ± 2	10 ± 1
C446P	24 ± 2	1 ± 0.1	100 ± 3	4 ± 1
C446S	23 ± 1	2 ± 0.1	164 ± 3	7 ± 1

B				
*Sa*AM	*K*_*m*_ (mg/ml)	*V*_*max*_ (µg CD min^−1^ µg protein^−1^)	*k*_*cat*_ (min^−1^) [10^3^]	*k*_*cat*_/*K*_*m*_(mg.ml^−1^ min^−1^) [10^3^]
WT	24 ± 6	0.08 ± 0.01	9 ± 1	0.35 ± 0.07
C446A	20 ± 5	0.08 ± 0.01	8 ± 1	0.42 ± 0.03
C446P	27 ± 8	0.06 ± 0.01	7 ± 1	0.25 ± 0.02
C446S	28 ± 7	0.07 ± 0.01	7 ± 1	0.28 ± 0.02

Data are shown as the mean ± standard deviation and are derived from three independent experiments.

The kinetic parameters for cyclization reaction were also investigated using pea starch as the substrate (Table 4B) The *K*_*m*_ values of WT and C446A were around 20–24 mg/ml, while those of C446P and C446S were 27–28 mg/ml, indicating that the P and S mutants had a lower affinity to pea starch than the WT. When compared with the WT, the catalytic rates of the P and S mutants were lower and the A mutant slightly higher. Thus, both binding of starch substrate and enzyme catalysis were affected upon C446 mutation to P and S. The change in substrate affinity suggested that the enzyme conformation was not optimal for catalysis. For proline, lacking an amide proton causes the P mutant the inability to form H-bond with side chain residues, thus, leading to protein destabilization^33^.

#### Analysis of LR-CD product pattern

**Figure 6:**
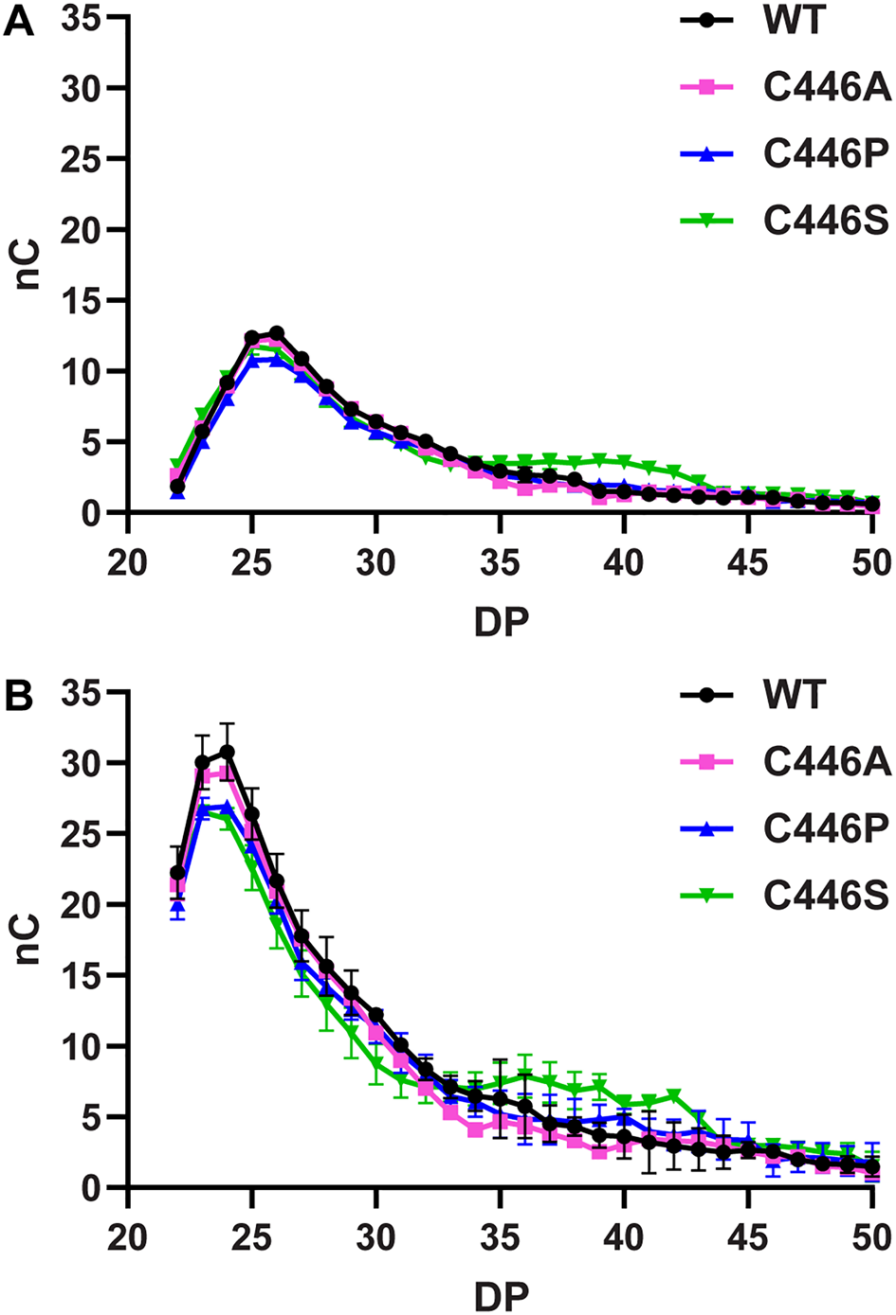
HPAEC-PAD analysis of LR-CD products synthesized at different incubation time: (**A**) 6 h and (**B**) 24 h, by WT and C446 mutated *Sa*AMs (*nC* signal response, *DP* Degree of polymerization).

Production of LR-CDs by cyclization activity of *Sa*AM was investigated. *Sa*AMs were incubated with 2% (w/v) pea starch at 30 °C and pH 6.0, which were their optimum conditions. LR-CD mixtures obtained were analyzed by HPAEC-PAD^41^. To compare the LR-CD patterns of WT and C446 mutated *Sa*AMs, the enzymes were incubated at various times. Unique LR-CD profiles of WT and C446 mutated *Sa*AMs in the range of CD22-CD50 were obtained with CD24-CD26 as main products and CD22 as the smallest product (Fig. 6A). Surprisingly, the product pattern of *Sa*AM was similar to those of the thermostable AMs, such as *Ta*AM^11,24^, but different from *Cg*AM of the mesophilic bacteria, which peaks at CD29-CD33^42^ with CD19 as the smallest^14^. We also found that the product pattern of larger LR-CD of the S mutant changed with time, i.e. more CD35-CD42 was produced at 24 h incubation than at 6 h (Fig. 6A,B), this is most probably due to their higher stability compared with the WT. At long incubation time, the yields of LR-CDs of all *Sa*AMs were significantly increased, with the pattern of principle LR-CD products shifted to the smaller size (CD23–CD24) at 24 h. The time dependence of product patterns observed was in accordance with that reported for *Cg*AM^14^.

From the overall biochemical characterization of the WT and mutated *Sa*AMs, the structure–function relationship could be explained in term of the obtained results. The importance of C446S in thermostability is evident from the 5 °C increase in optimum temperature and the threefold increase in its half-life time at 45 °C together with the increase in larger CD products observed at long incubation time. In addition, from structural analysis in Fig. 4, the constructed model of C446S based on the crystal structure of WT enzyme showed higher number of hydrogen bonds which is proposed to contribute to its thermostability.

### Structural basis for thermostability in AMs

In the attempt to analyze the molecular basis for thermostability in AMs, the structural data of different types of AM were compared (Table 5). As already mentioned, from the results of site-directed mutagenesis on optimum condition and thermostability of *Sa*AM, we proposed H-bonding contribution to enzyme thermostability, as supported by higher number of H-bonds in C446S mutants (Fig. 4). Apart from H-bonding between the functional groups of charged amino acid residues, other main factors that are known to stabilize proteins of hyperthermophiles are the number of ionic bonds and the presence of salt bridges^43^. It has been reported that the formation of salt bridges between nitrogen atom in guanidinium group of surface Arg with side chain carbonyl oxygen atoms of Asp/Glu within 4 Å and/or side chain-side chain H-bond within 3.5 Å are the interactions that play a major role in protein stability^44^. Analysis of amino acid sequences comparing *Sa*AM and thermophilic AMs revealed that *Thermus* has higher proline and hydrophobic amino acid composition (Table 5)^17,24^. However, because these *Thermus* are closely related species, amino acid composition analysis could be biased. This is corroborated by an observation that *A. aeolicus* AM (*Aa*AM)^12^, which is also thermophilic, has similar amino acid type composition (nonpolar, polar, and charged) compared with *Sa*AM. Thus, analysis of amino acid composition alone may not be sufficient to reveal the molecular basis for thermostability in AMs. Nonetheless, analysis of potential salt bridges and H bonds, based on the crystal structures showed that all thermophilic AMs have higher potential salt bridges; while the number of potential H bonds is more or less similar. When comparing *Sa*AM and *Aa*AM, which have similar amino acid type compositions, their number of H bonds are similar; but *Aa*AM has significantly a higher number of potential salt bridges. One previous report from our group on mutation of E27R showed that forming an Arg cluster R27-R30-R31-R34 on the enzyme surface resulted in a 10 °C increase in optimum temperature, hence, suggesting the contribution of salt bridge and H-bonding to thermostability of *Tf*AM^24^. Therefore, we could propose, from the results of our present study, that the increased salt bridges play a major role on thermostability of AMs.

**Table 5:** Amino acid compositions, number of potential salt bridges and H-bonds among AMs.

Amino acid	Amino acid composition (%) of amylomaltases				
	*S. agalactiae *(PDB ID 6M6T)	*T. aquaticus *(PDB ID 1ESW)^29^	*T. thermophilus *(PDB ID 2OWC)^19^	*T. brockianus *(PDB ID 2X1I)^18^	*A. aeolicus *(PDB ID 1TZ7)^20^
A	6.4	10.8	10.8	10.6	4.1
R	4.6	8.2	8.0	8.2	7.2
N	4.6	1.6	1.6	1.8	4.3
D	8.0	4.0	4.0	4.0	4.3
C	0.2	0.2	0.2	0.4	0.2
Q	4.0	2.0	2.2	2.2	1.4
E	8.0	10.0	10.0	10.4	11.8
G	6.8	9.0	9.0	9.0	6.4
H	1.6	2.6	2.6	2.6	1.9
I	6.6	2.2	2.2	2.8	4.3
L	7.6	10.8	10.8	10.8	12.4
K	6.2	3.4	3.4	2.6	8.0
M	2.8	2.0	2.0	1.6	1.0
F	5.8	6.2	6.2	6.4	7.4
P	4.0	7.6	7.6	7.2	4.9
S	4.0	2.8	2.8	3.0	4.1
T	6.4	3.0	3.0	3.4	2.3
W	2.8	4.2	4.2	4.4	3.1
Y	5.0	3.6	3.6	3.4	5.6
V	4.2	5.8	5.8	5.2	5.2
Nonpolar (G + A + V + L + M + I + F + Y + W + P)	52.0	62.2	62.2	61.4	54.4
Polar (S + T + C + N + Q)	19.2	9.6	9.8	10.8	12.3
Charged (K + R + H + D + E)	28.4	28.2	28.0	27.8	33.2
Number of potential salt bridges	236	295	272	283	337
Number of potential H bonds	261	288	276	271	264

## Conclusions

A novel amylomaltase from *S. agalactiae* showed high specific activities in the intermolecular transglycosylation (starch transglycosylation and disproportionation) and intramolecular transglycosylation (cyclization) reactions. Crystal structure determination confirmed its catalytic mechanism through the glycosyl-enzyme intermediate. In addition, we have captured the novel pre-transglycosylation conformation, which explains why the transglycosylation reaction is specific at the 4-OH. *Sa*AM is phylogenetically grouped with AMs from the mesophilic bacteria, but has a similar size to AMs of the thermophiles. It is, thus, a good model for studying thermostability in AMs through structural comparison and site-directed mutagenesis. From the analyses of properties of the mutated C446A/P/S, our results suggested the involvement of serine at the position 446 in the enhancement of thermal stability of *Sa*AM, as supported by a model structure showing an additional H-bonding with a nearby residue in the S mutant. Nevertheless, salt bridges might be more important than H-bonding in AM thermostability contribution, as evidenced by the results from the structural analysis.

## Materials and methods

### Bacteria, plasmid and chemicals

*Streptococcus agalactiae* FPrA02^45^ was kindly provided by Dr. Channarong Rodkhum of the Department of Veterinary Microbiology, Faculty of Veterinary Science, Chulalongkorn University, Bangkok. *E. coli* DH10 beta, restriction enzymes, DNA ligase, DNA polymerase and dNTPs were products of New England Biolabs Inc. (England). Quick-change kit was purchased from Stratagene (USA). pET-28a was from Novagen (USA). Plasmid purification kit was from Geneaid (Taiwan). HisTrap FF affinity column was from GE Healthcare (England). Standard oligosaccharides (G1–G7) and LR-CD were products of Wako Pure Chemical Industry Ltd. and Ezaki Glico (Japan), respectively. Glucose oxidase kit was from Human (Germany). Pea starch (Emsland-Starke GmbH, Germany) was kindly provided by Prof. Wolfgang Zimmermann of the University of Leipzig, Germany. All chemicals used were of analytical grade.

### Culturing of *S. agalactiae* and cloning of WT-AM gene

*S. agalactiae* was cultivated in brain heart infusion broth (HiMedia). The genomic DNA was extracted using the ZR Fungal/Bacterial DNA MiniPrep kit (Zymo Research). *AM* gene of *S. agalactiae* (*Sa*AM) was amplified by PCR technique with the primers 5′-TATACCATGGCTAAAAAACGTGCAAGTGGTGTCTTAAT-3′ and 5′-GGTGCTCGAGTTTATTCCCTCTATTATAAATAGTTGTAATCTCTTTTA-3′. The PCR product was then cut with *Nco*I and *Xho*I and cloned into the corresponding sites in pET28a. The nucleotide sequence was verified by sequencing to be identical to the previously reported sequence in the genome of *S. agalactiae* FPrA02^46^. The protein sequence is also identical to the entry WP_000745455 in GenBank.

### Amino acid sequence analysis, alignment and phylogeny

The multiple alignment of AMs was performed using ClustalW^47^; and the phylogenetic tree was constructed by a neighbour joining method^48^. Amino acid composition was analyzed by ProtParam^49,50^.

### Expression and purification of recombinant *Sa*AM

The recombinant *E. coli* cells containing *SaAM* gene were cultured in LB medium with 50 µg/ml of kanamycin. The cells were grown under constant shaking at 37 °C to OD_600_ 0.3–0.4. Then, expression of *Sa*AM was induced with 0.1 mM isopropylthio-β-d-galactoside (IPTG); and bacterial growth was continued with constant shaking for 3 h. Cells were collected by centrifugation (5000×*g*, 15 min at 4 °C) and suspended in 25 mM phosphate buffer pH 6.0. The collected cells were disrupted by sonication (15 cycles, 1 min each, on ice) and crude enzymes were obtained by centrifugation (20,000×*g*, 45 min at 4 °C). Both soluble and insoluble fractions were analyzed by SDS-PAGE. Crude enzymes were further purified by HisTrap FF column chromatography^14^. Protein bands were stained by Coomassie blue; and protein concentration was determined by Bradford method^51^. The purified fractions were pooled and assayed for AM activities.

### X-ray crystallography

The WT-*Sa*AM, that had been purified with Ni–NTA affinity chromatography, was dialyzed against 20 mM Tris pH 7.5. The protein solution was applied onto Q Sepharose and eluted with a linear gradient of 0–300 mM NaCl. Fractions containing pure WT-*Sa*AM were pooled, dialyzed against 20 mM Tris pH 7.5, concentrated to 40 mg/mL and supplemented with 10 mM acarbose. Crystallization was performed by microbatch under mineral oil by mixing 2 µL of protein with equal volume each of 100 mM Tris pH 7.5, 100 mM magnesium formate and 15% PEG 8000. The mixture was incubated at 16 °C. Crystals appeared and grew to full size within 3–4 days. The crystals were cryoprotected (in 100 mM Tris pH 7.5, 100 mM magnesium formate, 30% PEG 3350, and 10 mM acarbose), vitrified and stored in liquid nitrogen.

Single crystal diffraction data were collected at beamline BL13B1 of the National Synchrotron Radiation Research Center (Taiwan, Republic of China) (Table 1). Reflections were indexed and integrated using XDS^52^. Crystal symmetry determination and scaling were performed using AIMLESS^53^. Phaser^54^ was used for molecular replacement with the structure of *A. aeolicus* AM (PDB ID 1TZ7) as the search model (42.3% sequence identity to *Sa*AM). The model was built with PHENIX AutoBuild^55^. Refinement and model adjustment were performed with phenix.refine^56^ and COOT^57^, respectively. The structure was deposited at the Protein Data Bank under the accession code 6M6T. Structures were illustrated using PyMOL. Analyses of hydrogen bonds^58^ and salt bridges (charged atoms within 7 Å of each other) were performed using WHAT IF^59,60^.

### Construction of C446 mutated *Sa*AM gene

A recombinant plasmid (pET-28a) containing WT- *SaAM* was used as a template. The cysteine 446 (C446) residue of *Sa*AM was selected for site-directed mutagenesis. Replacements of C446 with three amino acids: alanine (A), proline (P) and serine (S), were performed. The mutated primers were shown in Table S1. PCR amplification technique was used to amplify C446 mutated *SaAM* genes. PCR conditions were: an initial denaturation at 98 °C for 30 s; followed by 15 cycles of amplification, each at 98 °C for 10 s, 55 °C for 30 s, and 68 °C for 4.5 min; and a post extension at 68 °C for 5 min. The pET-28a plasmids containing mutated *SaAM* were transformed into *E. coli* DH10 beta. The transformation colonies were selected on LB agar plate containing 50 µg/ml of kanamycin. After incubation at 37 °C for 16 h, the colonies were picked and cultured in LB medium containing 50 µg/ml of kanamycin for 12 h. The point mutation was confirmed by nucleotide sequencing and sequence alignment with Clustal W.

### Biochemical characterization

#### Assays for AM activity

The intermolecular transglycosylation (starch transglycosylation, disproportionation, coupling, and hydrolysis) and intramolecular transglycosylation (cyclization) activities of WT and mutated *Sa*AMs were determined at 30 °C or 40 °C as previously described^36,41^. In brief, starch transglycosylation activity was determined by measuring the reducing sugar released from starch and glucose substrates using the iodine method at 600 nm. For disproportionation, G3 was used as substrate; and glucose produced was determined by glucose oxidase method at 505 nm. Coupling activity was also measured by the glucose oxidase assay using LR-CDs and glucose as donor and acceptor substrates, respectively. The hydrolysis activity was determined by measuring the release of reducing sugar, as glucose, from LR-CDs substrate using the bicinchoninic acid assay at 562 nm. For cyclization activity, pea starch substrate was incubated with the enzyme for 1.5 h; then the reaction was stopped and glucoamylase was added and incubated for 12 h to hydrolyze linear oligosaccharides to glucose. The cyclic LR-CD product was detected by HPAEC-PAD and the unit enzyme was referred to the CA25 product. The mean values of all activities were calculated from three independent replicates.

#### Optimum conditions

The effects of pH and temperature on disproportionation activity of both WT and C446 mutated *Sa*AMs were determined. For optimum pH, the enzymes were incubated with 50 mM G3 in 50 mM buffers of different pH values (pH 3.0–9.0) at 40 °C for 10 min; then, the activities were respectively measured^36^. For optimum temperature, incubation was done in phosphate buffer pH 6.0 at various temperatures between 30 and 70 °C. The results were shown as percentage of relative activity. The highest activity was defined as 100%.

#### Temperature stability

The effect of temperature on stability of *Sa*AM was determined^36^. WT and C446 mutated *Sa*AMs were incubated in 50 mM phosphate buffer pH 6.0 at 30–50 °C for 0–180 min. Then, enzyme activities were measured. The results were presented as percentage of relative activity. The highest activity was defined as 100%.

#### Substrate specificity and kinetic studies

Malto-oligosaccharide substrates (G2–G7) were used for determining the specificity of *Sa*AM for disproportionation reaction^36^. 0.2 U of enzyme activity was incubated with 50 mM of substrate in phosphate buffer pH 6.0 at 40 °C for 10 min. The amount of glucose produced was determined by glucose oxidase method^41^. The relative activities were calculated by using WT activity with G3 substrate as control.

Kinetics of WT and C446 mutated *Sa*AMs were determined for disproportionation and cyclization reactions. For disproportionaion, incubations of various G3 substrate concentrations (0–40 mM) with 0.2 U of *Sa*AMs at optimum conditions were performed.

The enzyme activities were measured by glucose oxidase method^36^. For cyclization reaction, 0.5 U starch degrading activity of *Sa*AMs was incubated with various concentrations of pea starch substrate (0–0.5%) in 50 mM phosphate buffer pH 6.0, 30 °C for 1.5 h. LR-CD products were analyzed by HPAEC-PAD^41^. The kinetic parameters of WT and C447 mutated *Sa*AMs, *K*_*m*_ and *V*_*max*_, were determined from the non-linear analysis of the Michaelis–Menten equation. *k*_*cat*_ and *k*_*cat*_/*K*_*m*_ were also calculated.

#### Analysis of LR-CD product pattern

The patterns of LR-CD cyclization products of *Sa*AMs were investigated at different incubation times. The reaction mixtures, containing 0.2% (w/v) of pea starch and 0.1 U of enzymes, were incubated in 50 mM phosphate buffer pH 6.0 at 30 °C for 1.5, 6, 12, and 24 h. The LR-CD products were analyzed by HPAEC-PAD^41^.

## Supplementary Information

Supplementary Information.
