# Supplementary material for: Streptococcus agalactiae amylomaltase offers insight into the transglycosylation mechanism and the molecular basis of thermostability among amylomaltases
Source: Sci Rep. 2021 Mar 24;11:6740. doi: 10.1038/s41598-021-85769-3 (PMC7990933; doi:10.1038/s41598-021-85769-3)
Supplement: Supplementary file 1 — Supplementary Information. [file 41598_2021_85769_MOESM1_ESM.docx]

**Supplementary Information**

***Streptococcus agalactiae* amylomaltase offers insight into the transglycosylation mechanism and the molecular basis of thermostability among amylomaltases**

Suthipapun Tumhom^a^, Pitchanan Nimpiboon^a^, Kittikhun Wangkanont^b,c,*^, and Piamsook Pongsawasdi^a,*^

^a^Starch and Cyclodextrin Research unit, Department of Biochemistry, Faculty of Science, Chulalongkorn University, Bangkok 10330, Thailand

^b^Center of Excellence for Molecular Biology and Genomics of Shrimp, Department of Biochemistry, Faculty of Science, Chulalongkorn University, Bangkok 10330, Thailand

^c^Molecular Crop Research Unit, Department of Biochemistry, Faculty of Science, Chulalongkorn University, Bangkok 10330, Thailand

**^*^Corresponding authors**: kittikhun.w@chula.ac.th and piamsook.p@chula.ac.th

**Table S1.** C446 mutated *SaAM* gene primers for PCR mediated site-directed mutagenesis

| C446A_F | 5'accgttagtcaaacaacgattacagctatgcaagatctactagataaacc-3' |
| --- | --- |
| C446A_R | 5'ggtttatctagtagatcttgcatagctgtaatcgttgtttgactaacggt 3' |
| C446P_F | 5' accgttagtcaaacaacgattacacctatgcaagatctactagataaacc 3' |
| C446P_R | 5' ggtttatctagtagatcttgcataggtgtaatcgttgtttgactaacggt 3' |
| C446S_F | 5' ccgttagtcaaacaacgattacaagtatgcaagatctactagataa 3' |
| C446S_R | 5' ttatctagtagatcttgcatacttgtaatcgttgtttgactaacgg 3' |


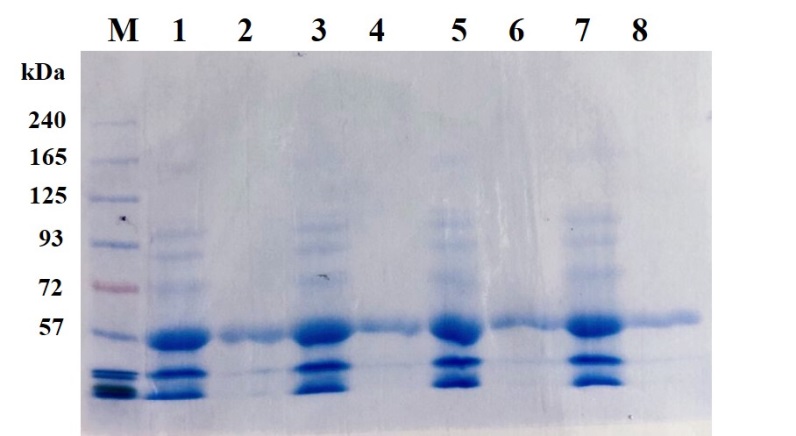


**Figure S1.** SDS PAGE analysis of the recombinant and C446 mutated amylomaltase from *S.* *agalactiae*. Lane M; protein molecular weight marker. Lane 1, 3, 5 and 7; 15 µg of crude enzyme of WT, C446A, P and S, respectively. Lane 2, 4, 6 and 8; 3 µg of purified enzyme of WT, C446A, P and S, respectively.


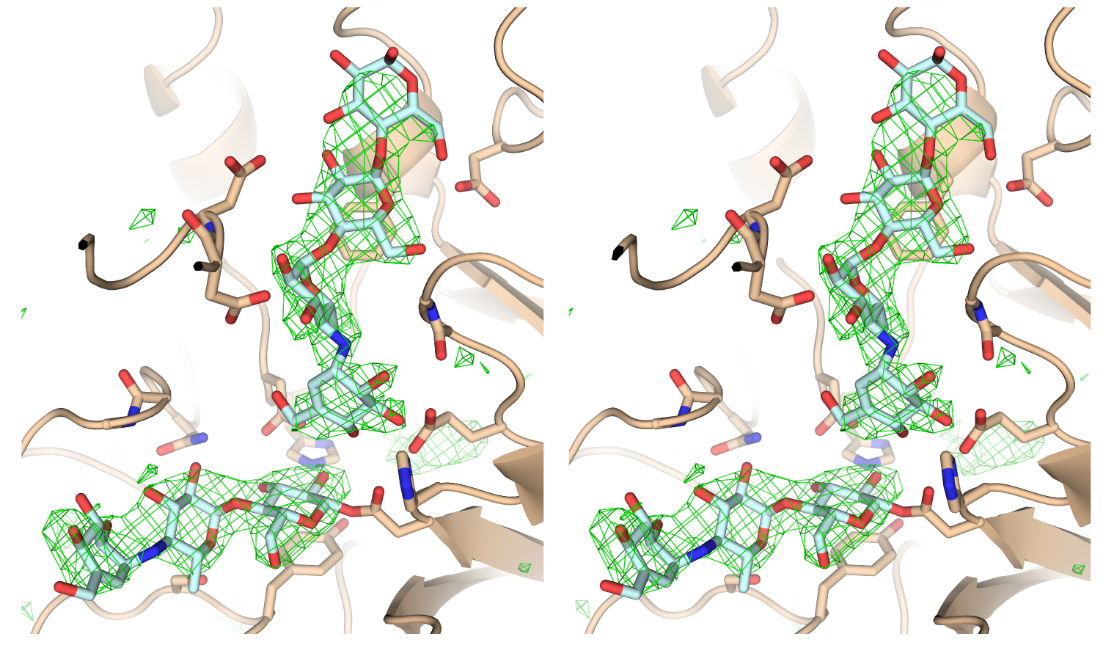


**Figure S2.** Stereo images of the *Sa*AM active site in the same pose as Fig 2 showing the mFo-DFc electron density map contoured at 3σ obtained after removal of all carbohydrate ligands and the structure re-refined with simulated annealing (5,000 to 300 K).
